# Effectiveness and safety of avelumab maintenance in patients aged ≥75 years with advanced urothelial cancer: a sub-analysis of the meet-URO 25 (MALVA) study

**DOI:** 10.3389/fimmu.2026.1818239

**Published:** 2026-06-11

**Authors:** Giandomenico Roviello, Elisabetta Gambale, Irene De Gennaro Aquino, Marco Maruzzo, Carlo Messina, Ismaela Anna Vascotto, Virginia Rossi, Davide Bimbatti, Elisa Erbetta, Marco Messina, Alessia Mennitto, Sara Elena Rebuzzi, Cecilia Nasso, Chiara Mercinelli, Martina Catalano, Brigida Anna Maiorano, Martina Fanelli, Mariella Sorarù, Federico Scolari, Marinella Micol Mela, Luca Galli, Alessia Salfi, Mimma Rizzo, Silvia Puglisi, Valentina Orlando, Giuseppe Fornarini, Alessandro Rametta, Patrizia Giannatempo, Linda Cerbone, Laura Doni, Serena Pillozzi, Lorenzo Antonuzzo

**Affiliations:** 1 Department of Health Sciences, Section of Clinical Pharmacology and Oncology, University of Florence, Florence, Italy; 2 Department of Experimental and Clinical Medicine, University of Florence, Florence, Italy; 3 Careggi University Hospital, Clinical Oncology, Florence, Italy; 4 Oncology 3 Unit, Department of Oncology, Istituto Oncologico Veneto IOV - IRCCS, Padova, Italy; 5 Ospedale Arnas Civico, Clinical Oncology, Palermo, Italy; 6 Medical Oncology 1 Unit, Department of Oncology, Istituto Oncologico Veneto IOV IRCCS, Padova, Italy; 7 Department of Surgery, Oncology and Gastroenterology, University of Padua, Padua, Italy; 8 Division of Oncology, University Hospital Maggiore della Carità, Novara, Italy; 9 Medical Oncology Unit 2, Ospedale Molinette Azienda Ospedaliero-Universitaria Città della Salute e della Scienza di Torino Corso Bramante 88, Torino, Italy; 10 Medical Oncology, Ospedale Santa Corona, Pietra Ligure, Italy; 11 Department of Medical Oncology, IRCCS San Raffaele Hospital, Comprehensive Cancer Center, and Vita-Salute San Raffaele University, Milan, Italy; 12 Medical Oncology Department, IRCCS San Raffaele Hospital, Milan, Italy; 13 Department of Oncology, University Hospital of Udine, Udine, Italy; 14 Ospedale di Camposampiero, U.O. Oncologia, Camposampiero, Italy; 15 Department of Biomedical, Experimental and Clinical Sciences, University of Florence, Florence, Italy; 16 Medical Oncology Unit 2, Azienda Ospedaliero-Universitaria Pisana, Pisa, Italy; 17 Oncologia Medica Universitaria Azienda Ospedaliera Universitaria Consorziale Policlinico di Bari piazza Giulio Cesare, Bari, Italy; 18 IRCCS Ospedale Policlinico San Martino, Genoa, Italy; 19 Department of Oncology, Ospedale Maggiore, Trieste, Italy; 20 Genitourinary Medical Oncology Department, Fondazione IRCCS Istituto Nazionale dei Tumori, Milan, Italy; 21 Medical Oncology 1, IRCCS National Cancer Institute Regina Elena, Rome, Italy

**Keywords:** avelumab, elderly patients, immunotherapy, maintenance therapy, real-world study, urothelial carcinoma

## Abstract

**Background:**

Avelumab maintenance therapy is an established therapeutic approach for patients with advanced urothelial carcinoma (UC) whose disease has not progressed after first-line platinum-based chemotherapy. However, evidence on its effectiveness and safety in elderly patients remains limited.

**Methods:**

This multicenter retro-prospective Italian study included 251 patients with advanced UC who received avelumab maintenance between 2021 and 2023. Outcomes were compared between patients aged ≥75 and <75 years. Primary endpoints were progression-free survival (PFS) and overall survival (OS).

**Results:**

Among 96 elderly and 155 younger patients, median PFS was 7.2 months (95% CI, 4.9–17.5) and 6.7 months (95% CI, 4.9–9.3), respectively (p = 0.547), while median OS was 28.1 months (95% CI, 17.3–42.1) versus 18.5 months (95% CI, 12.0–38.6) (p = 0.238). Immune-related adverse events were infrequent and generally comparable across age groups. In multivariable analyses, lung metastases independently predicted worse OS in elderly patients, whereas bone metastases and concomitant corticosteroid therapy were adverse prognostic factors in younger patients.

**Conclusions:**

Avelumab maintenance demonstrated similar efficacy and safety in elderly and younger patients, supporting its use regardless of age. In the evolving therapeutic landscape of advanced UC, this treatment strategy remains a relevant and practical option, particularly for elderly or comorbid patients who may not be candidates for more intensive first-line combinations.

## Introduction

Urothelial carcinoma (UC) represents the most common histological subtype of bladder cancer and accounts for more than 90% of all cases (1). Despite advances in systemic therapy, patients with locally advanced or metastatic UC continue to experience poor long-term outcomes, with a 5-year overall survival (OS) rate below 10% (1) (2),. Platinum-based chemotherapy has long represented the standard first-line treatment for eligible patients, providing initial disease control but limited durability of response (3). The JAVELIN Bladder 100 (4) trial established avelumab, an anti–PD-L1 monoclonal antibody, as the first maintenance therapy to significantly prolong both OS and progression-free survival (PFS) in patients with advanced UC whose disease had not progressed after first-line platinum-based chemotherapy. Based on these findings, avelumab maintenance has become a well-established therapeutic approach in this setting. More recently, the therapeutic landscape of advanced UC has rapidly evolved with the introduction of novel agents, including antibody–drug conjugates and immune checkpoint inhibitor combinations. In particular, the combination of enfortumab vedotin plus pembrolizumab has emerged as a new first-line standard of care in several settings (5). However, platinum-based chemotherapy followed by avelumab maintenance remains a possible and accessible strategy in routine clinical practice, especially for elderly or comorbid patients who may not be candidates for more intensive regimens (2). Unfortunately, real-world data describing the effectiveness and tolerability of avelumab, particularly in elderly or frail patients, who represent a large proportion of the UC population, remain limited.

Older patients frequently present with multiple comorbidities, impaired functional status, and reduced organ reserve, all of which may influence treatment decisions and outcomes (6, 7). In pivotal clinical trials, elderly individuals and those with poor performance status were often underrepresented (8), leading to uncertainty about the generalizability of trial results to routine clinical practice. Furthermore, advanced age is commonly associated with increased use of carboplatin-based regimens instead of cisplatin and with a higher risk of immune-related adverse events due to underlying comorbid conditions or polypharmacy (9, 10).

In this context, evaluating the real-world impact of avelumab maintenance therapy in older adults is of particular clinical relevance. A better understanding of safety, treatment duration, and survival outcomes in this population may help optimize patient selection and management in daily practice. The present retro-prospective subanalysis of the Meet-URO 25 MALVA study was designed to compare the efficacy and safety of avelumab maintenance therapy between elderly patients (≥75 years) and younger patients (<75 years) with advanced urothelial carcinoma, and to identify potential clinical predictors of survival in each subgroup. The choice of a 75-year age cut-off, rather than the more conventional 65-year threshold, was driven by the aim of identifying a truly elderly population more representative of real-world clinical practice, where patients aged ≥75 years often present distinct clinical and biological characteristics influencing treatment decisions and outcomes.

## Patients and methods

### Study design and population

This was a multicenter, retro-prospective observational study conducted across several oncology centers in Italy (11). In particular, this was a multicenter observational study including a retrospective cohort of patients treated before study initiation and a prospective cohort enrolled thereafter. The study included patients with advanced UC who achieved disease control after first-line platinum-based chemotherapy and subsequently received avelumab as maintenance therapy. A retrospective cohort of patients treated before formal study initiation was also incorporated to ensure comprehensive real-world representation.

Eligible participants were aged ≥18 years and had histologically or cytologically confirmed urothelial carcinoma with radiologic evidence of locally advanced or metastatic disease and no disease progression following 4–6 cycles of first-line platinum-based chemotherapy. Patients with both pure UC and variant histology were eligible for inclusion, provided that a predominant urothelial component was present. Patients who had previously received immune checkpoint inhibitors or who experienced progression during or immediately after chemotherapy were excluded. All participants provided written informed consent prior to data collection. Avelumab treatment was initiated between January 2021 and December 2023, and the data cutoff date for analysis was April 15, 2025.

### Data collection

Clinical and demographic data were extracted from institutional electronic medical records under local ethics committee approval. Variables included age, sex, primary tumor site, histology, metastatic sites, and prior local or systemic treatments. The type and number of chemotherapy cycles and response to prior treatment were also documented.

Comorbidities were recorded and categorized as cardiovascular, respiratory, genitourinary, metabolic (including diabetes and dyslipidemia), or autoimmune. A G8 geriatric screening score was collected when available to assess overall functional status and frailty.

The use of systemic corticosteroids, either before or during avelumab therapy, was documented independently of the indication (e.g., brain metastases, bone pain, fatigue, anorexia, chronic obstructive pulmonary disease (COPD), or autoimmune conditions).

### Treatment and assessment

All patients received avelumab 800 mg intravenously every two weeks as maintenance therapy until radiological or clinical progression, unacceptable toxicity, or withdrawal of consent. Tumor response was assessed according to RECIST version 1.1 criteria (12). Efficacy endpoints included OS and PFS as primary outcomes. OS was defined as the time from avelumab initiation to death from any cause, and PFS as the time from treatment start to documented progression or death. Objective response rate (ORR) (complete + partial response; (CR) and PR)) and disease control rate (DCR) (CR + PR + stable disease (SD)) were considered secondary endpoints. Patients without progression or death at the time of data cutoff were censored. Adverse events were retrospectively collected and graded according to the Common Terminology Criteria for Adverse Events (CTCAE) version 5.0.

### Statistical analysis

All analyses were performed using Stata SE version 18.0. Categorical variables were expressed as frequencies and percentages, and continuous variables as medians with ranges. Between-group comparisons (≥75 vs <75 years) were conducted using Chi-square or Fisher’s exact tests.

Survival outcomes were analyzed using the Kaplan–Meier method and compared using the log-rank test. Median follow-up was estimated via the reverse Kaplan–Meier method. Univariate and multivariate Cox proportional hazards models were used to evaluate prognostic variables for OS, and results were expressed as hazard ratios (HRs) with 95% confidence intervals (CIs). A *p*-value <0.05 was considered statistically significant.

An additional multivariable Cox proportional hazards model was performed in the overall study population to evaluate the independent prognostic role of age group (≥75 vs <75 years), adjusting for clinically relevant covariates including ECOG performance status, platinum-based chemotherapy regimen, metastatic disease at diagnosis, and the presence of bone and liver metastases.

The study protocol was reviewed and approved by and after by the Institutional Review Boards (IRBs) of all participating centers and conducted in accordance with the Declaration of Helsinki and Good Clinical Practice (GCP) guidelines, (trial registration number: MALVA study; CEAVC 20817_oss; Comitato Etico Regionale per la Sperimentazione Clinica della Regione Toscana: Sezione: AREA VASTA CENTRO; Careggi University of Florence Hospital ethical committee). All patients provided written informed consent for participation and data collection. Patient confidentiality was maintained throughout the study, and all data were anonymized before analysis.

## Results

### Patient characteristics

A total of 251 (62 (24.7%) enrolled retrospectively and 189 (75.3%) prospectively) patients treated with avelumab maintenance therapy were included in the analysis: 96 patients (38.2%) were aged ≥75 years, and 155 (61.8%) were younger than 75 years. Baseline demographic and clinical characteristics are summarized in **Table 1**.

**Table 1:** Patient baseline characteristics.

Variables	≥75 years number (96)	<75 years number (155)	P
Age, median (range), years	79 (75-88)	67 (38-74)	<0.01
Sex, n (%)			0.866
Male	78 (81.2)	128 (82.6)	
Female	18 (18.8)	27 (17.4)	
ECOG performance status, n (%)			0.003
0	49 (51.0)	103 (66.6)	
1	46 (47.9)	43 (27.7)	
≥2	1 (1.1)	9 (5.7)	
Primary tumor site, n (%)			0.765
Bladder	71 (73.9)	115 (74.2)	
Upper urinary tract	25 (26.1)	40 (25.8)	
Hystology			0.815
Urothelial	89 (92.7)	141 (91)	
Variant	7 (7.3)	14 (9.0)	
Site of metastasis, n (%)			
Lung	29 (30.2)	49 (31.6)	0.815
Bone	19 (19.8)	47 (30.3)	0.066
Liver	11 (11.5)	24 (15.5)	0.371
Lymph node	79 (82.3)	113 (72.9)	0.088
Brain	2 (2.1)	1 (0.6)	0.560
Other	21 (21.9)	24 (15.5)	0.236
Metastatic at diagnosis n (%)			0.001
Yes	29 (30.2)	79 (51.0)	
No	67 (69.8)	76 (49.0)	
Prior treatment, n (%)			
Surgery	49 (51.0)	60 (38.7)	0.067
Radio chemotherapy	1 (1.1)	1 (0.7)	o.615
Prior chemotherapy regimen, n (%)			<0.001
Gemcitabine + cisplatin	26 (27.1)	91 (59.1)	
Gemcitabine + carboplatin	70 (72.9)	63 (40.9)	
Number of cycles of prior chemotherapy, n (%)			0.584
4	72 (75.0)	107 (69.0)	
5	7 (7.3)	16 (10.3)	
6	17 (17.7)	32 (20.7)	
Best overall response to prior chemotherapy, n (%)			0.406
CR	5 (5.2)	7 (4.5)	
PR	56 (58.3)	78 (50.3)	
SD	35 (36.5)	70 (45.2)	

Median age was 79 years (range 75–88) in the elderly group and 67 years (range 38–74) in younger patients (p<0.01). The proportion of male and female patients was similar across age groups (81.2% vs 82.6%, p=0.866).

Patients aged ≥75 years more frequently presented with an ECOG performance status ≥1 compared with younger patients (49.0% vs 33.4%, p=0.003). The primary tumor site (bladder vs upper urinary tract) and histological subtype (pure urothelial vs variant) did not differ significantly between the two groups.

Lymph node metastases were common in both cohorts (82.3% vs 72.9%), whereas *de novo* metastatic disease was more frequent among younger patients (51.0% vs 30.2%, p=0.001). Elderly patients were more often treated with gemcitabine plus carboplatin as first-line chemotherapy (72.9% vs 40.9%, p<0.001), reflecting the influence of age and comorbidities on treatment selection. No significant differences were observed in the number of chemotherapy cycles or best response to prior chemotherapy (**Table 1**).

### Comorbidities and geriatric status

As shown in **Table 2**, comorbidities were highly prevalent in elderly patients. Cardiovascular diseases (77.1% vs 58.1%, p=0.002) and respiratory disorders (13.5% vs 5.2%, p=0.032) were significantly more common in patients aged ≥75 years, while diabetes, dyslipidemia, and autoimmune diseases occurred at similar percentages. G8 score was available in 42 of 96 elderly patients (43.7%) and in 46 of 155 younger patients (29.7%). Among evaluable patients, the median G8 score was 13 in both groups (p=0.476). The median G8 score was 13 in both groups (p=0.476), suggesting that most elderly patients retained a good functional status and were appropriate candidates for maintenance immunotherapy.

**Table 2:** Patient’s comorbidities according age.

Comorbidities, n (%)	≥75 years number (96)	<75 years number (155)	P
Cardiovascular	74 (77.1)	90 (58.1)	0.002
Respiratory	13 (13.5)	8 (5.2)	0.032
Genitourinary	8 (8.3)	15 (9.7)	0.824
Diabetes	16 (16.7)	28 (18.1)	0.865
Dyslipidaemia	25 (26.0)	39 (25.2)	0.883
Autoimmune disease	6 (6.2)	8 (5.2)	0.780
≥ 3 comorbidities	11 (11.5)	19 (12.3)	0.849
G8 score median (range)	13 (9-17) *Available for* *42 (43.7%)*	13 (8-17) *Available for 46 (29.7%)*	0.476

### Avelumab-related toxicity

**Figure 1:**
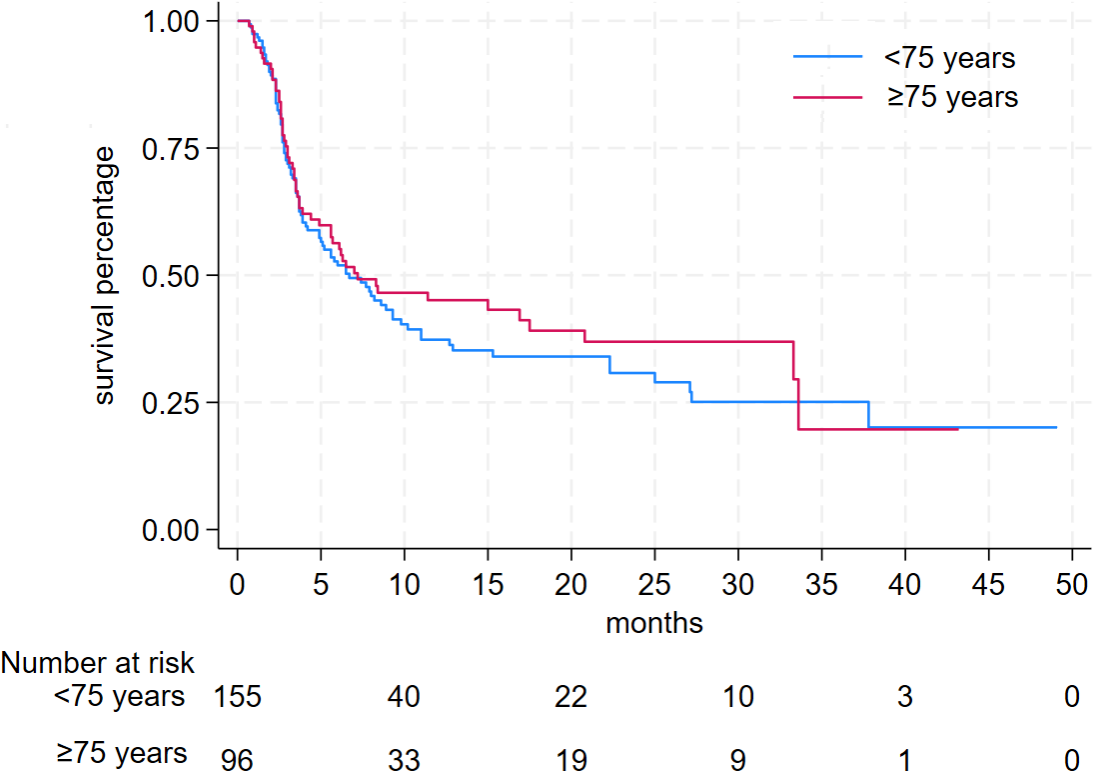
Progression-free survival (PFS) according to age group.

The overall safety profile of avelumab was comparable across age groups (**Table 3**: **Figure 1**). Early immune-related toxicity occurred in 7.3% of elderly and 5.8% of younger patients (p=0.791). The rate of hospitalization due to toxicity was low in both groups (3.1% vs 3.9%, p=1.000).

**Table 3:** Most relevant toxicity from avelumab according age.

Variables	≥75 years number (96)	<75 years number (155)	P
Early toxicity from avelumab	7 (7.3)	9 (5.8)	0.791
Hospitalisation from avelumab toxicity	3 (3.1)	6 (3.9)	1.000
Intestinal toxicity	4 (4.2)	7 (4.5)	1.000
Hepatic toxicity	0	3 (2.0)	0.289
Skin toxicity	4 (4.2)	10 (6.5)	0.576
Lung Toxicity	1 (1.0)	1 (0.66)	1.000
Pancreatic toxicity	1 (1.0)	2 (1.3)	1.000
Neurologic Toxicity	0	2 (1.3)	0.526
Pituitary toxicity	1 (1.0)	1 (0.66)	1.000
Thyroid Toxicity	11 (11.5)	12 (7.7)	0.370
Other toxicity	14 (14.6)	19 (12.3)	0.701
Delayed immune-related adverse events	2 (2.1)	2 (1.3)	0.638
Deaths probably related to avelumab	1 (1.6)	2 (1.9)	1.000
Steroid use before to start avelumab	5 (5.2)	14 (9.0)	0.331
Steroid use during avelumab	16 (16.7)	18 (11.6)	0.261

Thyroid toxicity represented the most commonly reported immune-related adverse event in both groups (11.5% vs 7.7%), followed by skin and intestinal toxicity, without statistically significant differences. Severe events, such as hepatic, pulmonary, or neurological toxicity, were rare (<3%). Only one death was considered probably related to avelumab in the elderly group (1.6%) and two deaths in the younger group (1.9%).

The use of steroids before starting avelumab was slightly more frequent in younger patients (9.0% vs 5.2%), while steroid use during treatment tended to be higher among the elderly (16.7% vs 11.6%), though the difference was not statistically significant (p=0.261).

### Treatment duration and subsequent therapies

As summarized in **Table 4**, treatment discontinuation occurred in 59.4% of elderly and 67.1% of younger patients (p=0.215). Disease progression was the leading cause of discontinuation (82.5% vs 79.8%), followed by treatment-related toxicity (14.0% vs 17.3%).

**Table 4:** Treatment discontinuation and subsequent therapy according age.

Variables	≥75 years number (96)	<75 years number (155)	P
Avelumab maintenance therapy ongoing, n (%) Avelumab maintenance therapy discontinued, n (%)	39 (40.6) 57 (59.4)	51 (32.9) 104 (67.1)	0.215
Reason for discontinuation, n (%) Disease progression Toxicity* Unknow Other *Including three deaths probably related to avelumab	57 (59.4) 47 (82.5) 8 (14.0) 1 (1.7) 1 (1.7)	104 (67.1) 83 (79.8) 18 (17.3) 3 (2.9) 0	0.619
Subsequent therapy (main)	39 (40.6)	55 (35.5)	0.413

The proportion of patients who received subsequent systemic therapy after discontinuing avelumab was comparable between age groups (40.6% vs 35.5%, p=0.413). This suggests that chronological age alone did not limit access to further treatments after immunotherapy.

### Efficacy outcomes

Objective response rates to avelumab maintenance therapy are reported in **Table 5**. The ORR was 29.5% in patients aged ≥75 years and 25.0% in younger patients (p=0.539). The DCR was similar between groups (70.4% vs 68.6%, p=0.764).

**Table 5:** Best response, PFS and OS according to age.

	All patients (N = 251)	≥75 years number (96)	<75 years number (155)	P
RC	25 (9.96)	13 (13.5)	12 (7.7)	0.685
PR	36 (14.34)	13 (13.5)	23 (14.8)	
SD	97 (38.65)	36 (37.6)	61 (39.6)	
PD	70 (27.89)	26 (27.1)	44 (28.4)	
UNK	23 (9.16)	8 (8.3)	15 (9.7)	
RR (RC+PR)	61 (26.75)	26 (29.5)	35 (25.0)	0.539
DCR (RC+PR + SD)	158 (69.30)	62 (70.4)	96 (68.6)	0.764
PFS M-months (95% IC)	7 (5.6-9.3)	7.2 (4.9-17.5)	6.7 (4.9-9.3)	0.547
OS M-months (95% IC)	22.4 (16.5-36.6)	28.1 (17.3-42.1)	18.5 (12.0-38.6)	0.238

**Figure 2:**
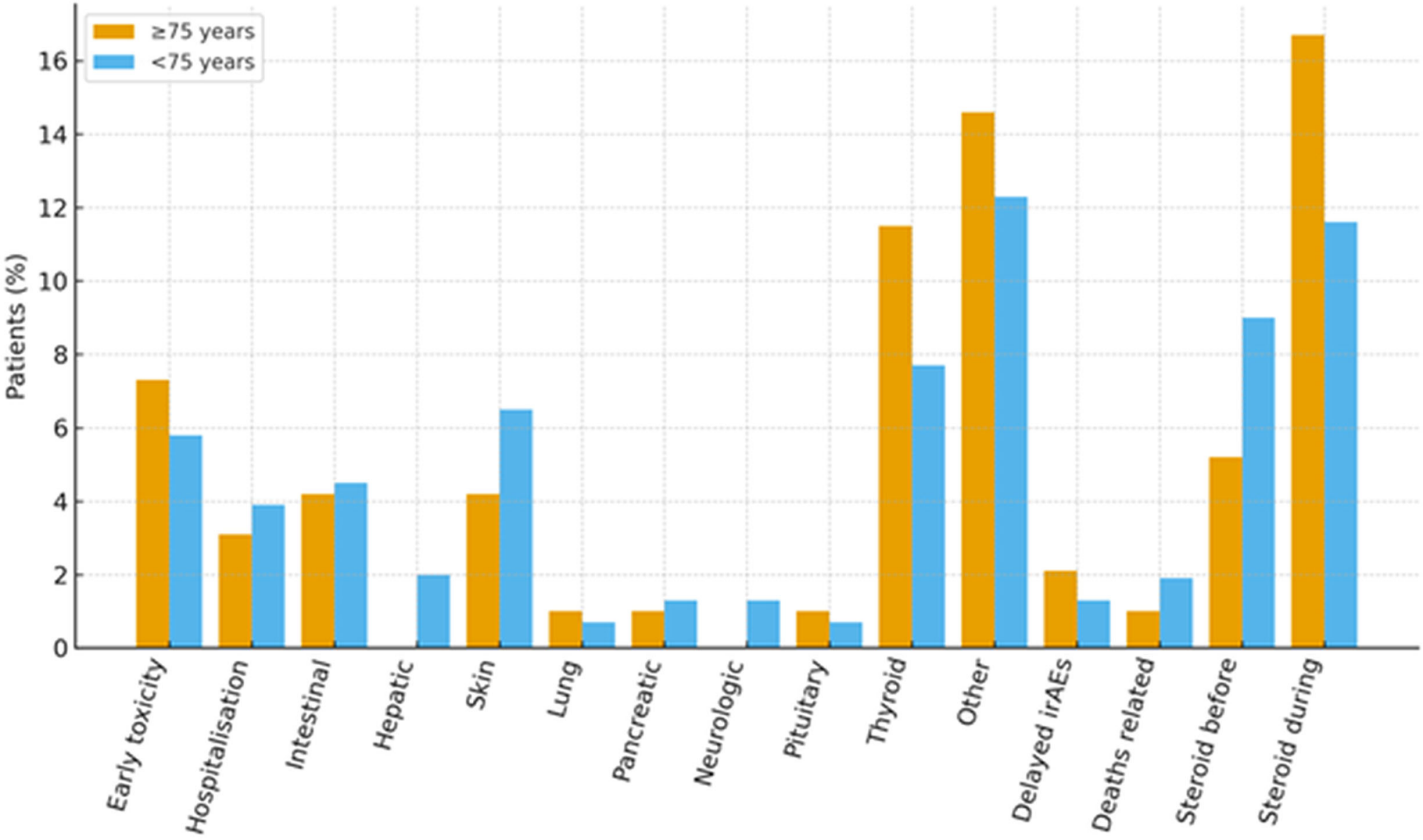
Incidence (%) of Avelumab-related toxicities in older (≥75 years) versus younger (<75 years) patients.

**Figure 3:**
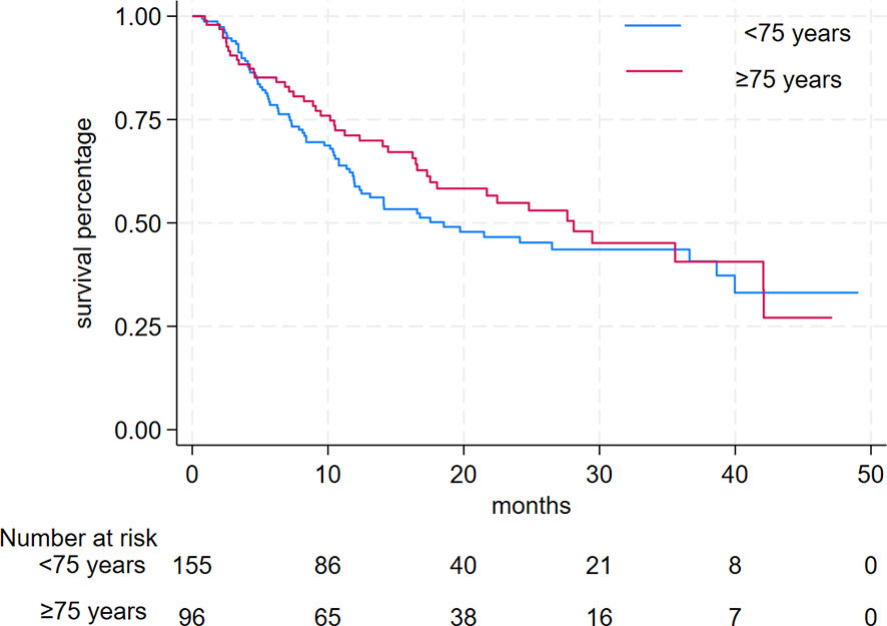
Overall survival (OS) according to age group.

Median progression-free survival (PFS) was 7.2 months (95% CI: 4.9–17.5) in elderly patients and 6.7 months (95% CI: 4.9–9.3) in younger patients (p=0.547; **Figure 2**). Median OS tended to be longer in the elderly group (28.1 months, 95% CI: 17.3–42.1) compared with younger patients (18.5 months, 95% CI: 12.0–38.6), though the difference was not statistically significant (p=0.238; **Figure 3**).

### Univariate and multivariable survival analyses

In the univariate analysis (**Table 6**), several factors were associated with poorer OS. Among elderly patients, ECOG performance status ≥1 (HR 1.89, 95% CI, 1.03–3.47; p = 0.040), lung metastases (HR 2.09, 95% CI, 1.15–3.78; p = 0.015), liver metastases (HR 2.36, 95% CI, 1.08–5.15; p = 0.030), brain metastases (HR 14.5, 95% CI, 2.99–70.55; p = 0.001), and concomitant steroid therapy (HR 4.04, 95% CI, 1.55–10.50; p = 0.004) were associated with shorter survival. In the younger group, adverse prognostic factors included ECOG performance status ≥1 (HR 2.10, 95% CI, 1.30–3.38; p = 0.002), diabetes (HR 1.69, 95% CI, 1.00–2.85; p = 0.049), ≥3 comorbidities (HR 1.85, 95% CI, 1.00–3.46; p = 0.050), bone metastases (HR 2.93, 95% CI, 1.83–4.68; p < 0.001), concomitant steroid therapy (HR 2.87, 95% CI, 1.44–5.71; p = 0.003), and steroid use during avelumab treatment (HR 2.15, 95% CI, 1.15–4.00; p = 0.016).

**Table 6:** Univariate for OS according age.

	≥75 years number (96)			<75 years number (155)		
	HR	IC 95%	P	HR	IC 95%	P
Ecog (≥1 vs 0)	1.89	1.03-3.47	**0.040**	2.10	1.30-3.38	**0.002**
Sex (male vs female)	1.18	0.56-2.48	0.652	0.79	0.40-1.55	0.508
Cardiovascular comorbidity (yes vs no)	0.96	0.48-1.91	0.920	1.00	0.62-1.59	0.998
Respiratory comorbidity (yes vs no)	0.86	0.33-2.19	0.759	0.83	0.26-2.65	0.761
Genitourinary comorbidity (yes vs no)	0.84	0.25-2.73	0.773	1.30	0.62-2.72	0.479
Diabetes (yes vs no)	0.61	0.26-1.46	0.273	1.69	1.00-2.85	**0.049**
Dyslipidaemia (yes vs no)	1.00	0.50-1.99	0.999	1.19	0.69-2.07	0.514
≥ 3 comorbidities (yes vs no)	0.74	0.26-2.08	0.574	1.85	1.00-3.46	**0.050**
Autoimmune disorder (yes vs no)	5.59	–	1.000	0.93	0.29-2.97	0.905
Previous neoadjuvant cht (yes vs no)	0.86	0.11-6.33	0.890	2.08	0.83-5.19	0.115
Previous surgery (yes vs no)	0.92	0.51-1.68	0.804	1.44	0.90-2.28	0.120
Site of primary tumor (lower vs upper)	0.89	0.75-1.56	0.821	0.72	0.45-1.10	0.121
Histology (urothelial vs variants)	1.29	0.31-5.38	0.721	0.87	0.41-1.81	0.712
Metastatic at diagnosis (yes vs no)	1.20	0.62-2.32	0.572	0.93	0.58-1.48	0.761
Chemotherapy (cis+gem vs Carbo+gem)	0.87	0.45-1.71	0.706	2.08	0.83-5.19	0.115
Cycles I line chemotherapy (>4 vs 4)	0.83	0.41-1.69	0.625	1.11	0.68-1.80	0.672
Response I line chemotherapy (RC-PR vs SD)	1.17	0.63-2.18	0.615	1.43	0.94-2.19	0.089
Lung metastasis (yes vs no)	2.09	1.15-3.78	**0.015**	0.74	0.45-1.22	0.243
Lymph node metastasis (yes vs no)	0.54	0.27-1.06	0.075	0.98	0.59-1.63	0.953
Bone metastasis (yes vs no)	1.58	0.79-3.14	0.188	2.93	1.83-4.68	**<0.001**
Liver metastasis (yes vs no)	2.36	1.08-5.15	**0.030**	1.22	0.65-2.27	0.529
Brain metastasis (yes vs no)	14.5	2.99-70.55	**0.001**	2.52	–	1.000
Other metastasis (yes vs no)	0.93	0.44-1.94	0.856	1.44	0.81-2.55	0.203
Concomitant steroid therapy (yes vs no)	4.04	1.55-10.50	**0.004**	2.87	1.44-5.71	**0.003**
Steroid use during Avelumab (yes vs no)	1.44	0.70-2.95	0.311	2.15	1.15-4.00	**0.016**

Bold values mean statistically significant.

In the multivariable analysis (**Table 7**), brain metastases were not included in the model because of the very limited number of events, which could have generated unstable estimates and model overfitting. Among patients aged ≥75 years, lung metastases remained independently associated with worse OS (HR 2.29, 95% CI, 1.24–4.42; p = 0.008). In patients aged <75 years, bone metastases (HR 2.40, 95% CI, 1.44–3.99; p = 0.001) and concomitant steroid therapy (HR 2.30, 95% CI, 1.13–4.67; p = 0.021) retained independent prognostic significance.

**Table 7:** Multivariate for OS according age.

	≥75 years number (96)		
	HR	IC 95%	P
Ecog (≥1 vs 0)	1.84	0.96-3.55	0.066
Lung metastasis (yes vs no)	2.29	1.24-4.42	**0.008**
Liver metastasis (yes vs no)	1.80	0.77-4.22	0.172
Concomitant steroid therapy (yes vs no)	2.38	0.88-3.23	0.123
	<75 years Number (155)		
Ecog (≥1 vs 0)	1.48	0.89-2.47	0.127
Diabetes (yes vs no)	1.44	0.79-2.63	0.231
≥ 3 comorbidities (yes vs no)	1.37	0.66-2.81	0.390
Bone metastasis (yes vs no)	2.40	1.44-3.99	**0.001**
Concomitant steroid therapy (yes vs no)	2.30	1.13-4.67	**0.021**
Steroid use during Avelumab (yes vs no)	1.60	0.84-3.06	0.150

Bold values mean statistically significant.

### Prognostic analyses in the overall study population

To further investigate the independent prognostic impact of age, additional univariate and multivariable Cox proportional hazards analyses for OS were performed in the overall study population (**Table 8**). In the univariate analysis, ECOG performance status ≥1 (HR 1.86, 95% CI 1.29–2.67; p=0.001), bone metastases (HR 2.44, 95% CI 1.67–3.56; p<0.001), and brain metastases (HR 15.45, 95% CI 3.51–68.01; p<0.001) were associated with worse OS, while age group (≥75 vs <75 years) was not significantly associated with outcome (HR 0.82, 95% CI 0.56–1.19; p=0.301).

**Table 8:** Univariate Cox proportional hazards analysis for overall survival in the overall study population.

Variable	HR	95% CI	p-value
Sex (male vs female)	0.93	0.57–1.53	0.789
Metastatic at diagnosis (yes vs no)	1.07	0.73–1.55	0.740
Chemotherapy (cis+gem vs Carbo+gem)	1.10	0.76–1.59	0.611
Lymph node metastasis (yes vs no)	0.80	0.54–1.20	0.289
Bone metastasis (yes vs no)	2.44	1.67–3.56	<0.001
Liver metastasis (yes vs no)	1.57	0.97–2.54	0.068
Lung metastasis (yes vs no)	1.11	0.76–1.62	0.573
Brain metastasis (yes vs no)	15.45	3.51–68.01	<0.001
Age ≥75 years (yes vs no)	0.82	0.56–1.19	0.301
Previous surgery (yes vs no)	1.18	0.82–1.70	0.369
Ecog (≥1 vs 0)	1.86	1.29–2.67	0.001
Histology (urothelial vs variants)	0.94	0.49–1.80	0.860

A multivariable Cox regression model including age group, ECOG performance status, platinum-based chemotherapy regimen, metastatic disease at diagnosis, and major metastatic sites was subsequently performed (**Table 9**). Brain metastases were not included in the multivariable model because of the very limited number of events, which could have generated unstable estimates and potential model overfitting. In the adjusted analysis, ECOG performance status ≥1 (HR 1.62, 95% CI 1.08–2.43; p=0.019) and bone metastases (HR 2.05, 95% CI 1.37–3.07; p<0.001) remained independently associated with worse OS. Conversely, age group was not independently associated with survival outcomes (HR 0.77, 95% CI 0.51–1.17; p=0.216), supporting the comparable efficacy of avelumab maintenance therapy across age groups after adjustment for baseline prognostic factors.

**Table 9:** Multivariable Cox proportional hazards analysis for overall survival in the overall study population.

Variable	HR	95% CI	p-value
Age ≥75 years (yes vs no)	0.77	0.51–1.17	0.216
Ecog (≥1 vs 0)	1.62	1.08–2.43	0.019
Chemotherapy (cis+gem vs Carbo+gem)	1.12	0.74–1.68	0.588
Metastatic at diagnosis (yes vs no)	1.02	0.67–1.54	0.943
Bone metastasis (yes vs no)	2.05	1.37–3.07	<0.001
Liver metastasis (yes vs no)	1.28	0.76–2.16	0.351

## Discussion

In this large multicenter real-world analysis, we investigated the efficacy and safety of avelumab first-line maintenance therapy in elderly patients with advanced UC compared with a younger population. Our findings indicate that biological age does not significantly influence treatment outcomes, and that elderly patients, despite presenting with more comorbidities and a higher rate of impaired performance status, achieve similar benefits in terms of progression-free and OS. The results confirm that maintenance immunotherapy with avelumab is both feasible and effective in routine clinical practice, even in patients aged 75 years or older. Notably, the choice of a cut-off at 75 years, although relatively high compared with conventional age stratifications used in oncologic studies, reflects the demographic reality of patients with urothelial carcinoma and aligns with real-world clinical practice, in which this threshold more accurately distinguishes the truly elderly population.

The outcomes observed in our cohort are consistent with those reported in pivotal and real-world studies. In the phase III JAVELIN Bladder 100 trial (5), which established avelumab as the standard maintenance approach, Gupta et al. (13) recently published an age-specific analysis showing preserved efficacy across subgroups: median OS reached 26.1 months in patients aged ≥65 years, 24.0 months in those ≥75 years, and 24.9 months among individuals ≥80 years, compared with 15.5, 13.5, and 10.0 months, respectively, in the best supportive care group. Importantly, no new safety concerns emerged with increasing age, and the relative benefit of avelumab was maintained across all categories. These findings align with our own results, where the elderly cohort achieved an OS of 28.1 months and PFS of 7.2 months, values comparable or even slightly superior to those from the pivotal trial.

Additional confirmation derives from the large real-world AVENANCE (14) study conducted in France, which enrolled 595 patients with a median age of 73 years. Despite including a population more heterogeneous and frail than that of JAVELIN Bladder 100^5^, median OS was 21.3 months and PFS 5.7 months, with grade ≥3 immune-related adverse events reported in fewer than 10% of patients. Interestingly, among those who received subsequent enfortumab vedotin, median OS extended beyond 40 months, highlighting the importance of treatment sequencing after avelumab. Similarly, the RAVE-Bladder ambispective study (15) confirmed the reproducibility of these outcomes in clinical practice, reporting a 1-year OS of 78.7% and median PFS of 9.5 months in a cohort with a median age of 65 years (range 40–84). Collectively, these datasets confirm that avelumab maintains robust efficacy and favorable tolerability regardless of age or baseline fitness, supporting its use in older and comorbid patients.

In our study, treatment discontinuation and toxicity profiles were comparable between age groups. Immune-related adverse events were generally mild, with thyroid and cutaneous reactions being most common, and severe events remained uncommon (<3%). Only isolated treatment-related deaths occurred in either group, reflecting the overall good tolerability of avelumab in daily practice. The slightly higher frequency of corticosteroid use among elderly patients, mainly for comorbidity management, did not translate into higher discontinuation rates or toxicity. Nonetheless, as also seen in previous reports, steroid exposure correlated with poorer survival, likely reflecting underlying disease burden rather than a direct pharmacologic effect (16). Taken together, these findings demonstrate that the safety of avelumab is not substantially affected by age, corroborating the results from the JAVELIN Bladder 100^5^ age subgroup analysis and from AVENANCE (14), both of which showed no excess of immune-related or severe adverse events among elderly individuals.

When exploring prognostic factors, performance status and metastatic pattern emerged as major determinants of outcome, consistent with previous evidence (11). In the present cohort, lung metastases were independently associated with worse prognosis among elderly patients, whereas bone metastases and concomitant corticosteroid therapy emerged as adverse prognostic factors in the younger population. Although brain metastases were associated with poor outcomes in exploratory analyses, this finding should be interpreted with caution due to the very limited number of patients and the consequent instability of the estimates. These results mirror those reported by Furubayashi et al. (17), who found that baseline ECOG performance status and visceral disease, but not age, significantly influenced post-avelumab outcomes. Overall, our findings reinforce that functional status and metastatic burden, rather than chronological age, should guide therapeutic decision-making. Additionally, we observed that the elderly population included in this study represents a relatively fit and selected subgroup, as suggested by the preserved G8 score despite a higher proportion of ECOG ≥1. This may partly explain the numerically longer OS observed in patients aged ≥75 years. These findings should therefore be interpreted with caution, as they likely reflect a selection bias toward fitter elderly individuals eligible for both platinum-based chemotherapy and maintenance immunotherapy, reflecting the “fit elderly” selection bias, a well-recognized phenomenon in real-world immunotherapy cohorts. Another relevant aspect is the significant difference in the type of platinum-based chemotherapy administered between age groups. Elderly patients were more frequently treated with carboplatin-based regimens compared with younger individuals, reflecting differences in cisplatin eligibility driven by comorbidities and functional status. This imbalance may represent a potential confounding factor in the interpretation of survival outcomes, as treatment selection in routine clinical practice is inherently influenced by patient characteristics (“confounding by indication”). Patients receiving carboplatin are generally considered less fit and may have a worse baseline prognosis. Nevertheless, despite the higher use of carboplatin in the elderly group, survival outcomes were comparable between age categories, suggesting that avelumab maintenance may help overcome, at least partially, baseline differences related to first-line treatment selection. Finally, in our cohort, younger patients more frequently presented with metastatic disease at diagnosis compared with elderly individuals. This difference may reflect a more aggressive disease biology or a higher likelihood of delayed diagnosis in younger patients, although alternative explanations such as referral patterns and selection biases cannot be excluded. Importantly, despite this imbalance, survival outcomes remained comparable between age groups, further supporting the notion that age alone does not significantly influence the effectiveness of avelumab maintenance therapy.

In the current therapeutic landscape, the positioning of platinum-based chemotherapy followed by avelumab maintenance deserves careful consideration. Although the combination of enfortumab vedotin plus pembrolizumab is redefining first-line treatment (5), this approach may not be suitable for all patients, particularly the elderly and those with significant comorbidities. In this context, chemotherapy followed by avelumab maintenance remains a clinically relevant treatment strategy supported by both randomized clinical trials and real-world evidence, including the present study. Our findings suggest that this approach is feasible and generally well tolerated in elderly patients treated in routine clinical practice. In addition, emerging real-world evidence suggests that sequential treatment strategies incorporating immunotherapy and antibody–drug conjugates may be associated with prolonged survival outcomes (18). Overall, avelumab maintenance continues to represent a reasonable therapeutic option within the evolving treatment landscape of advanced urothelial carcinoma.

This study has several limitations, including its retrospective design and potential selection bias toward fitter elderly individuals. Moreover, geriatric assessment tools such as the G8 score were not systematically available for all patients. However, the multicenter nature of the cohort, the inclusion of over 250 patients, and the consistent methodology provide robust support for the external validity of these findings.

In conclusion, our data confirm that avelumab first-line maintenance offers comparable efficacy and safety in elderly and younger patients with advanced UC. Chronological age should not be used as an exclusion criterion, as older patients with preserved performance status and manageable comorbidities can derive substantial benefit from maintenance immunotherapy. These observations, in agreement with the JAVELIN Bladder 100, AVENANCE, and RAVE-Bladder studies, highlight the pivotal role of avelumab in the continuum of care for advanced UC and emphasize the need for individualized, function-based rather than age-based, treatment strategies in this growing population.

## Data Availability

The raw data supporting the conclusions of this article will be made available by the authors, without undue reservation.

## References

[1] Siegel RL Kratzer TB Giaquinto AN Jemal A Wagle NS Miller KD . Cancer statistics, 2025. CA Cancer J Clin. (2025) 75:10–45. doi: 10.3322/caac.21871 39817679 PMC11745215

[2] Roviello G Santoni M Sonpavde GP Catalano M . The evolving treatment landscape of metastatic urothelial cancer. Nat Rev Urol. (2024) 21:580–92. doi: 10.1038/s41585-024-00872-0 38702396

[3] Azam F Alharbi H Alshangiti A Zar Gul AR Bukhari N Ouda M . Eligibility criteria for different platinum-based chemotherapy regimens in metastatic urothelial carcinoma. Cureus. (2024) 16:e66520. doi: 10.7759/cureus.66520 39246966 PMC11380918

[4] Powles T Park SH Voog E Caserta C Valderrama BP Gurney H . Avelumab maintenance therapy for advanced or metastatic urothelialcarcinoma. N Engl J Med. (2020) 383:1218–30. doi: 10.1056/NEJMoa2002788 32945632

[5] Powles T Valderrama BP Gupta S Bedke J Kikuchi E Hoffman-Censits J . Enfortumab vedotin and pembrolizumab in untreated advanced urothelial cancer. N Engl J Med. (2024) 390:875–88. doi: 10.1056/NEJMoa2312117 38446675

[6] Woolford SJ Sohan O Dennison EM Cooper C Patel HP . Approaches to the diagnosis and prevention of frailty. Aging Clin Exp Res. (2020) 32:1629–37. doi: 10.1007/s40520-020-01559-3 32356135 PMC7508740

[7] Aggarwal P Woolford SJ Patel HP . Multi-morbidity and polypharmacy in older people: challenges and opportunities for clinical practice. Geriatrics (Basel). (2020) 5:85. doi: 10.3390/geriatrics5040085 33126470 PMC7709573

[8] van Marum RJ . Underrepresentation of the elderly in clinical trials, time for action. Br J Clin Pharmacol. (2020) 86:2014–6. doi: 10.1111/bcp.14539 32909294 PMC7495271

[9] Fotopoulou C . Limitations to the use of carboplatin-based therapy in advanced ovarian cancer. EJC Suppl. (2014) 12:13–6. doi: 10.1016/S1359-6349(15)70005-4 26759527 PMC4683379

[10] Esen BH Bektas SN Topcu U Köylü B Kuvvet FBB Bahat G . Immune-related adverse events in older adults receiving immune checkpoint inhibitors: a comprehensive analysis of the Food and Drug Administration Adverse Event Reporting System. Age Ageing. (2025) 54:afaf008. doi: 10.1093/ageing/afaf008 39883592 PMC11781319

[11] Roviello G Gambale E Aquino IG Maruzzo M Messina C Vascotto IA . Real-world effectiveness of avelumab maintenance in advanced urothelial carcinoma: results from the Italian multicenter MALVA study (Meet-URO 25). Oncologist. (2025) 30:oyaf388. doi: 10.1093/oncolo/oyaf388 41264480 PMC12680435

[12] Schwartz LH Litière S de Vries E Ford R Gwyther S Mandrekar S . RECIST 1.1 – update and clarification: from the RECIST committee. Eur J Cancer. (2016) 62:132–7. doi: 10.1016/j.ejca.2016.03.081 27189322 PMC5737828

[13] Gupta S Climent Duran MA Sridhar SS Powles T Bellmunt J Park SH . Avelumab first-line maintenance for advanced urothelial carcinoma: long-term outcomes from the JAVELIN Bladder 100 trial in older patients. ESMO Open. (2025) 10:104506. doi: 10.1016/j.esmoop.2025.104506 40107155 PMC11964637

[14] Barthélémy P Thibault C Fléchon A Gross-Goupil M Voog E Eymard JC . Real-world study of avelumab first-line maintenance treatment in patients with advanced urothelial carcinoma in France: overall results from the noninterventional AVENANCE study and analysis of outcomes by second-line treatment. Eur Urol Oncol. (2025) 8:407–16. doi: 10.1016/j.euo.2024.09.014 39448350

[15] Tsimafeyeu I Gridneva Y Sultanbaev A Anzhiganova Y Gluzman M Mochalova A . Maintenance therapy with avelumab for patients with metastatic urothelial carcinoma: a real-world, ambispective RAVE-Bladder study. Cancer Med. (2025) 14:e70636. doi: 10.1002/cam4.70636 39950261 PMC11826084

[16] Petrelli F Signorelli D Ghidini M Ghidini A Pizzutilo EG Ruggieri L . Association of steroids use with survival in patients treated with immune checkpoint inhibitors: A systematic review and meta-analysis. Cancers (Basel). (2020) 12:546. doi: 10.3390/cancers12030546 32120803 PMC7139305

[17] Furubayashi N Mochida M Kijima A Fujimoto Y Nakamura M Negishi T . Outcomes of avelumab treatment in advanced urothelial carcinoma according to age, performance status, and timing. In Vivo. (2025) 39:976–87. doi: 10.21873/invivo.13903 40010984 PMC11884485

[18] Fiala O Massari F Basso U Giannatempo P Grande E Buti S . Enfortumab vedotin following platinum chemotherapy and avelumab maintenance in patients with metastatic urothelial carcinoma: a retrospective data from the ARON-2(EV) study. Target Oncol. (2024) 19:905–15. doi: 10.1007/s11523-024-01099-0 39354179 PMC11557677

